# What can the citations of systematic reviews of ethical literature tell us about their use?—an explorative empirical analysis of 31 reviews

**DOI:** 10.1186/s13643-023-02341-y

**Published:** 2023-09-23

**Authors:** Hélène Nobile, Natali Lilie Randjbar Moshtaghin, Zoë Lüddecke, Antje Schnarr, Marcel Mertz

**Affiliations:** 1 Institute for Ethics, History and Philosophy of Medicine, Hannover Medical School, Carl-Neuberg-Str. 1, 30625 Hannover, Germany; 2 Institute for the History of Medicine and Medical Ethics, Faculty of Medicine and University Hospital, University of Cologne, Joseph-Stelzmann-Str. 20, Geb. 42, 50931 Cologne, Germany

**Keywords:** Systematic review, Literature review, Meta-research, Ethics, Citation practice

## Abstract

**Background:**

Systematic reviews of ethical literature (SREL) aim at providing an overview of ethical issues, arguments, or concepts on a specific ethical topic. As SREL are becoming more common, their methodology and possible impact are increasingly subjected to critical considerations. Because they analyse and synthetise normative literature, SREL are likely to be used differently than typical systematic reviews. Still, the uses and the expected purposes of SREL were, to date, mainly theoretically discussed. Our explorative study aimed at gaining preliminary empirical insights into the actual uses of SREL.

**Methods:**

Citations of SREL in publications, both scientific and non-scientific, were taken as proxy for SREL uses. The citations of 31 published SREL were systematically searched on Google Scholar. Each citation was qualitatively analysed to determine its function. The resulting categorisation of SREL citations was further quantitatively investigated to unveil possible trends.

**Results:**

The analysis of the resulting sample of SREL citations (n=1812) showed that the selected SREL were mostly cited to support claims about ethical issues, arguments, or concepts, but also to merely mention the existence of literature on a given topic. In this sample, SREL were cited predominantly within empirical publications in journals from various academic fields, indicating a broad, field-independent use of such systematic reviews. The selected SREL were also used as methodological orientations either for the conduct of SREL or for the practical and ethically sensitive conduct of empirical studies.

**Conclusions:**

In our sample, SREL were rarely used to develop guidelines or to derive ethical recommendations, as it is often postulated in the theoretical literature. The findings of this study constitute a valuable preliminary empirical input in the current methodological debate on SREL and could contribute to developing strategies to align expected purposes with actual uses of SREL.

**Supplementary Information:**

The online version contains supplementary material available at 10.1186/s13643-023-02341-y.

## Background

Systematic reviews (SR) of scientific literature are secondary research studies that aim at the objective synthesis of all available published evidence on a given scientific topic [1]. In order to ensure the quality, objectivity and readability of the results obtained in such studies, guidelines have been developed, thereby standardising the research and reporting processes, i.e. search, selection, analysis and synthesis (e.g. “Preferred Reporting Items for Systematic Reviews and Meta-Analyses” (PRISMA) [2]). The results generated by SR constitute crucial inputs for evidence-based medicine, guidelines development or health technology assessments (HTA) [3].

The heterogeneity of the scientific and scholarly literature requires certain adjustments of the SR method according to specific disciplines. Natural sciences, including medical investigations, on the one hand, tend to generate numerical data that can be statistically aggregated in meta-analyses. Literature belonging to social sciences, on the other hand, can produce non-numerical qualitative data that require alternative reviewing approaches. Ethical literature, by contrast, often consists of theoretical normative content, e.g., discussing ethical issues, evaluating practices and processes, or judgments about the ethical outcomes of a course of action. However, it can also comprise empirical qualitative or quantitative investigations, the results of which are then used, for example, either as sources of ethical arguments or as descriptions of ethical issues.

Systematic Reviews of Ethical Literature (SREL) aim at the comprehensive and systematically structured overview of appropriate literature in the light of its relevance for normative questions, e.g., ethical issues, reasons/arguments or concepts on a specific question. The question of the adequate method to reach this goal has been recurring in the field of bioethics [4, 5]. As the number of SREL steadily increased over the past three decades [6–8], a wide lexical variety has developed: SR “of argument-based (ethics) literature” [9–11]; SR “of reasons” [12, 13]; SR “of normative (bioethics) literature” [4, 14]; SR “for normative evidence” [15]; or “ethics syntheses” [16, 17]. This heterogeneity is representative of the debates around SR within the bioethics community. While some have challenged the accuracy or even the legitimacy of the expression “SR” for reviews of ethical literature [5], others have questioned the suitability or even the necessity of the SR method in this field [14]. Conversely, there have been different calls to adapt the “classical” SR methodology to standardise this alternative approach for literature analysis and synthesis [9, 12, 14]. As a result, guidelines are currently being developed specifically for SREL, “PRISMA-Ethics” [18].

Despite these methodological specificities, SREL remains an application of the general SR methodology. So there is a natural assumption to consider the impact of SREL similar to the one obtained by “traditional” SR, i.e., being a valuable input for clinical decision making, guideline development or HTA. Up to now, the possible impact of SREL has exclusively been debated on a theoretical level [5, 9, 14, 19], and lacks an empirical foundation. Implementing an empirical, qualitative or quantitative, investigation of the actual uses of SREL bears many challenges, not least in the question of deciding on a suitable sample of possible SREL users. An alternative promising empirical approach could be found in the field of “citationology” where citations are used to detect the signs authors give as they refer or cite previously published literature [20]. Such citation analysis proceeds to the careful analysis of the context in which citations occur, in order to unveil the different meanings and trends in citing practices [21].

We therefore decided to perform an exploratory empirical study to investigate the actual uses of SREL through their citations. To this end, we designated citations of published SREL as proxy for the actual SREL uses. We thus conducted a systematic tracking and analysis of SREL citations, beginning with a qualitative investigation of their nature and then proceeding to a quantitative study of their frequencies. With this mixed design, we aim to provide preliminary answers to the following two-fold research question: how (for what purpose) and where (i.e., type of literature, text section, kind of journal) are SREL cited?

## Methods

To determine our sample of SREL from which we analysed the citations, we used the results of a systematic search we implemented in a previous meta-review (*n* = 51 SREL, originally designated as “SR of normative literature”) [6]. Because of the time span required for a publication to enter a citation cycle, we refrained from searching the literature for more recent SREL. Within this initial sample of SREL, we decided to focus on reviews published from 2010 on (*n* = 33) in order to comply with the available resources. Since we decided to work with SREL written in one of the languages all authors could analyse, i.e., English and German, we excluded 1 SREL written in French.

This process eventually resulted in a final sample of 32 SREL, published from 2010 until 2015. In preparation for the main study, we performed a pilot phase with one of the selected SREL in which we tested the steps described below to test their feasibility (AS, MM). This stage resulted in slight revisions to improve procedures.

### Citing publications: search

First, we conducted a citation search of all included SREL on the web search engine Google Scholar where the number of citations, i.e., documents containing a least one citation of the SREL, is provided for each entry. These documents are in the following called “citing publications” in order to distinguish them from the actual citations within a specific publication. Each citing publication is listed once in Google Scholar even when the citing publication entails more than one citation of the SREL of interest. We decided to use Google Scholar as it was shown to perform well in terms of the number of citations retrieved, as compared to other search engines of databases such as Scopus or Web of Science [22]. It further appeared to be an effective way to retrieve a broad range of document types, and not only scientific publications as it would have been the case on a database like PubMed. Since our goal was not to retrieve full samples of citations for each selected SREL, we did not proceed to further searches on other search engines or databases.

Secondly, we attempted to retrieve the full-texts of all citing publications. At this stage, we could detect that some entries found on Google Scholar had to be excluded because they did not entail a citation from the SREL of interest. We thus set as a rule that the actual number of citations can only be determined after full-text checks. At this stage and at the time of our search, Google Scholar indicated that one SREL had not been cited. As the absence of citation made it impossible to analyze this SREL according to our criteria, we excluded it, which left a final sample of 31 SREL. The citation search was performed by one of the authors (NR) and took place between February and July 2020.

### Citing publications: selection

Once the citing publications of each selected SREL were retrieved, we proceeded with a two-step selection procedure. A first selection was performed based on language, accessibility, and publication status. To be included, citing documents had to be (a) written either in English or German; (b) accessible online, openly or through our institutional credentials, i.e., Hannover Medical School; and (c) published, including scientific as well as non-scientific publications. The second selection was based on a full-text screening of the documents, including reference lists and supplements. At this stage, citing publications were excluded if (a) they did not mention the SREL of interest in the reference list; (b) if they did not cite the SREL in the text; or (c) if they were duplicates of already selected citing publications. Quality appraisal was not used as a criterion for selection: all citing documents that remained after the two-step selection were included in the analysis. An overview on the inclusion and exclusion criteria is provided in Table 1.

**Table 1:** Inclusion/exclusion criteria: two-step selection of the citing publications

1st step selection: Inclusion criteria	Publication	All document types, provided they are published
	Language	Documents written in English or German only
	Access	Documents accessible online (open or through institutional credentials)
2nd step selection: Exclusion criteria	SREL citation	No reference of the SREL of interest in the reference list
		No citation of the SREL in the text
	Duplicates	

### Citation extraction from citing publications

To answer our research question, we collected all citations of the SREL of interest within each included citing publication of the 32 selected SREL.

### Analysis

Each step of the analysis described in this section was performed independently by at least two researchers involved in this study.

#### Systematic reviews of ethical literature

To unveil potential trends in the citation process, we selected a set of variables related to the specificities of the SREL under consideration. Next to the year of publication and country of origin of the first author, we documented the four following variables for each SREL: (1) topic; (2) object of review; (3) academic field of the publishing journal; (4) presence and nature of recommendations.

The *topics* of the selected SREL were independently analysed by two of the authors (HN, MM), then discussed and categorised using a combination of inductive and deductive strategies, as already performed in one of our previous publications [8].

The *objects of review* of each selected review were retrieved and classified (HN, MM) following the definition of information units the authors detailed in a previous publication [6] and presented in Table 3. The categories for the information units were the following: (1) Ethical issues, topics, dilemmas; (2) Ethical arguments, reasons; (3) Ethical principles, values, concepts; (4) Ethical guidelines, recommendations; (5) Other.

For each included SREL, we determined the *academic field of the journal* in which the review was published based on their classification in the *Journal Citation Report* [23], using mainly the *Science Citation Index Expanded* and the *Social Sciences Citation Index*. In case these two indexes did not classify the publishing journal, we used alternative indexes, namely the *Emerging Sources Citation Index* and the *Arts & Humanities Citation Index*. This classification was performed by two of the authors (HN and MM).

Lastly, we analysed whether the selected SREL did include *recommendations*. If recommendations were issued, we distinguished between two types of recommendations: (1) recommendation of a practical nature that suggest ethically informed changes or improvements in practice, e.g., care, research planning, informed consent; (2) recommendation that suggests changes or improvements in aspects different than practice such as methodology, future research development, teaching material to improve the ethical value of their expected outcomes or authorship decision. It has to be noted here that the quality of the recommendation was not assessed in the frame of our analysis. The sole fact that authors used formulations that clearly indicated a recommendation was considered decisive for classification. This classification was performed by two of the authors (HN and MM).

#### Citations

During the pilot phase mentioned above, we trialed our preliminary coding frame that was constituted by a set of variables chosen for their expected ability to unveil possible citation patterns. This trial led to further discussion of the codes resulting in improvements and refinements within the coding frame. The pilot testing was undertaken by two authors (AS and MM). Next to authors’ name and publication year, the following characteristics were determined for each citing publication: (a) document type; (b) citation type; (c) localisation of the citation in case the document used the common scientific structure, i.e., Introduction, Methods, Results, and Discussion (IMRaD); (d) direct quotation.

To classify the *document type*, we proceeded with a strategy combining inductive and deductive approaches. A major distinction was made between publications with a scientific format and publications with other formats. Within the publications with a scientific format, we distinguished: (1) methodological; (2) conceptual or philosophical); (3) empirical; (4) review, e.g., narrative or scoping; (5) systematic review; (6) comment or letter; (7) editorial; (8) scientific report (defined as written documents with standards equivalent to those of scientific publications but published on organisation websites only, e.g., HTA, WHO, national ethics councils). Dissertations and Master theses were classified according to the nature of their main goal, e.g., empirical or conceptual investigation. Among the publications with non-scientific formats, we distinguished between: (1) blog; (2) newspaper article; (3) guidelines, recommendations; (4) decision aids; (5) teaching material; (6) material for self-help; (7) patient information; (8) conference proceedings; (9) other. Classification of document types was performed by 2 authors (ZL and HN, partly with the support of an intern) and, in case of uncertainties, discussed with a third author (MM).

To analyse the *citation type*, we conducted a qualitative oriented category-based content analysis [24]) i.e., we qualitatively analysed the nature of the citations in order to categorise them and then use this categorisation for our subsequent quantitative investigation. In this qualitative part of the assessment, we first distinguished citations with a methodological scope from those with a thematic significance. In each of these two categories, we then evaluated the extent to which the citation was used in the publication. The coding frame was first developed on the theoretical basis of the authors’ scholarly experience regarding citations and their functions, and then inductively supplemented through our pilot testing. Our categories are similar to an already existing functional categorisation, i.e., “negative” (“refuted”), “perfunctory” (“noted only”), “compared”, “used” and “substantiated” ([25] as cited by [20]). During the main analysis, we maintained the possibility to further inductively expand the coding frame. All citations were classified within the coding frame by one author (NR), and each citation was double-checked by at least another author (AS or MM). At the beginning, about a third of all citing publications were triple checked (MM checked NR’s coding as well as AS’s double-check), and remaining uncertainties were discussed among the three authors, in order to refine coding rules and together gain familiarity with the material and with solving ambiguous cases. An overview about the citations classification as well as some examples is presented in Table 2.

**Table 2:** Categorisation of the citations from systematic reviews of ethical literature: definitions and examples

CITATIONS WITH A METHODOLOGICAL OR A THEMATIC SCOPE		
**Category**	**Definition**	**Examples**
Mention	The citation is merely mentioned to point out the existence of publications developing a method or addressing a topic. The text does not use, explain, or detail the citation or its content further (perfunctory)	*Patients and families carry similar misconceptions about palliative care, often perceiving PPC [Patient Priorities Care] as a type of EOL [End of Life] care implemented only when curative cancer treatments have been exhausted and children and families have no other hope *[reference to SREL [26]]. Extracted from citing publication [27]
Support	The citation is linked to concrete content regarding the method or the topic, but its content is neither discussed nor transformed. It is only used as a reference to support, substantiate or justify a specific statement	*All research involving living beings calls for deliberate ethical considerations. When conducting research with children, the ethical stakes are viewed as higher, due to their need of protection [reference to SREL* [28]]. Extracted from citing publication [29]
SREL as research object	The review is taken as a research object by the citing publication. The cited SREL constitutes part of the evaluated data of the citing publication e.g., in an overview of the SR methods or in an overview of the issues related to a topic	*As shown in *Fig. 3*, initial database extractions resulted in 235 citations, which were narrowed down to n* = *88 articles retrieved directly from databases (and n* = *12 retrieved from additional hand searches resulting in N* = *100 total articles) meeting inclusion/exclusion criteria after deduplication, title/abstract, and full-text review [final sample included SREL * [30]]. Extracted from citing publication [31]
Analysis /Transformation	The citing publication works with detailed content-related statements or methodological arguments from the SREL to formulate its own statements or arguments. This generally includes content transformation such as comparison, appreciation, or criticism	*I suspect that one of the primary reasons why the definition of moral distress is often confined to constraint-distress is because of moral concerns about the “additional” burden of distress that might be experienced by nurses [reference to SREL* [32]*]. Any health professional could suffer distress due to concerns about patient care, which can lead to the well-being of that professional being negatively impacted. In these kinds of cases, only the moral values of patient well-being and professional well-being are likely to be violated.* Extracted from citing publication [33]
Recommendation	Recommendations or requirements are derived from or supported by statements or arguments originating from the quoted SREL. These recommendations or guidances go beyond the methodological or thematic content of the cited review	*Technically, a sham operation is the most appropriate control and should be considered the gold-standard control for future studies, however the ethics of this need to be considered [reference to SREL* [34]*]*. Extracted from citing publication [35]
Constitutive for citing publication	The citation is constitutive for the research question or the methodology of the citing publication, for example to distinguish one’s own research question from the quoted SREL	*Although transcriptions differ among the various nations, a valid basis for international protocol acceptance has been set. Nevertheless, there are discrepancies among the diverse transcriptions. The whole area is still in a constant move and many of the regulations and guidelines still lack clarity or are difficult to access [reference to SREL* [36]*]. Therefore, selection of a suitable protocol template was one of the crucial points of this work*. Extracted from citing publication [37]
Reading suggestion	The SREL is merely referenced as a bibliographical recommendation	*In light of these criticisms, some ethicists have advanced alternatives to the best interests standard, such as Diekema’s (2004) “harm principle” (for an overview, refer to [reference to SREL* [38]*].* Extracted from citing publication [39]
CITATIONS WITH A METHODOLOGICAL SCOPE ONLY		
Positive appropriation / Adoption	The citation of SREL methods is used by the citing publication for developing, justifying or modifying its own methods, or for comparing it to its own methods	*Recognizing that ‘state-of-the-art’ reviews can be valuable in steering researchers towards more rigorous and useful research practice [reference to SREL* [30]*], we undertook a systematic, thematic literature review to characterize recent approaches to examining age-related differences in receipt of healthcare and public health interventions*. Extracted from citing publication [40]
Negative appropriation / reutilisation	The citing publication uses the citation to demarcate its own method from the method mentioned in the cited SREL, or else to develop or justify a different method	*More specific subthemes were identified within the subject Benefits and Risks (*Table 3*, in bold in columns 2 and 3); these were used to classify the benefits and risks and the labels were derived and modified from the classification outlined by Ayuso *et al*., 2013 [reference to SREL* [41]*].* Extracted from citing publication [42]

**Table 3:** General characteristics of the selected 31 systematic reviews of ethical literature

Year	Author(s)	Country	Title	Publishing journal (*JAF*)	Review topic	Object of review	Inclusion of recommendations
2015	Kalkman et al. [43]	NL	*Pragmatic randomized trials in drug development pose new ethical questions: a systematic review*	Drug Discovery Today (*Pharmacology & Pharmacy*)	Research Ethics: Clinical trial	Ethical issues	No
	McCarthy et al. [32]	IE	*Moral distress: a review of the argument-based nursing ethics literature*	Nursing Ethics (*Nursing*)	Clinical Ethics: Nursing	Ethical arguments	No
	Niemansburg et al. [34]	NL	*Reconsidering the ethics of sham interventions in an era of emerging technologies*	Surgery (*Surgery*)	Research Ethics: Clinical trial	Ethical arguments	Recommendations for research
	Preshaw et al. [44]	UK	*Ethical issues experienced by healthcare workers in nursing homes: literature review*	Nursing Ethics (*Nursing*)	Clinical Ethics: Geriatrics	Ethical issues and principles	No
2014	Calvert et al. [36]	UK	*Patient-reported outcome (PRO) assessment in clinical trials: a systematic review of guidance for trial protocol writers*	PloS One (*Multidisciplinary sciences*)	Research Ethics: Clinical trial	Ethical recommendations	Recommendations for research
	Huang et al. [28]	AU	*Ethical and methodological issues in qualitative health research involving children: A systematic review*	Nursing Ethics (*Nursing*)	Research Ethics: Paediatric research	Ethical issues and arguments	Recommendations for practice
	Jamshidi et al. [45]	IR	*Ethical Considerations of Community-based Participatory Research: Contextual Underpinnings for Developing Countries*	International Journal of Preventive Medicine (*Medicine, General and internal)*	Research Ethics: Community-based participatory research	Ethical issues and guidelines	No
	McDougall and Notini [38]	AU	*Overriding parents’ medical decisions for their children: a systematic review of normative literature*	Journal of Medical Ethics (*Medical Ethics*)	Clinical Ethics: Pediatric care	Ethical recommendations	No
	Pratt et al. [46]	US	*Perspectives from South and East Asia on clinical and research ethics: a literature review*	Journal of Empirical Research on Human Research Ethics (*Medical Ethics*)	Clinical and Research Ethics	Ethical values	No
	Valdez-Martinez et al. [26]	MX	*When to stop? Decision-making when children's cancer treatment is no longer curative: a mixed-method systematic review*	BMC Pediatrics (*Pediatrics*)	Clinical Ethics: Pediatric care	Ethical recommendations and issues	Recommendations for research
	Van der Dam et al. [47]	NL	*Ethics support in institutional elderly care: a review of the literature*	Journal of Medical Ethics (*Medical Ethics*)	Clinical Ethics: Geriatrics	Ethical recommendations	No
	Schildmann and Schildmann [48]	DE	*Palliative sedation therapy: a systematic literature review and critical appraisal of available guidance on indication and decision making*	Journal of Palliative Medicine (*Health care sciences and services*)	Clinical Ethics: End of Life	Ethical recommendations	No
	Whicher et al. [49]	US	*Ethical Issues in Patient Safety Research: A Systematic Review of the Literature*	Journal of Patient Safety (*Healthcare science and service*)	Research Ethics: Safety	Ethical issues and arguments	No
2013	Ayuso et al. [41]	SP	*Informed consent for whole-genome sequencing studies in the clinical setting. Proposed recommendations on essential content and process*	European Journal of Human Genetics (*Genetics and Heredity*)	Research Ethics: Informed consent	Ethical issues and recommendations	Recommendations for practice
	Christenhusz et al. [50]	BE	*To tell or not to tell? A systematic review of ethical reflections on incidental findings arising in genetics contexts*	European Journal of Human Genetics (*Genetics and Heredity*)	Research Ethics: Genetics	Ethical reasons	Recommendations for practice
	Mikesell et al. [30]	US	*Ethical community-engaged research: a literature review*	American Journal of Public Health (*Public, Environmental and Occupational Health*)	Research Ethics: Community-based participatory research	Ethical principles and issues	No
	Strech et al. [51]	DE	*The full spectrum of ethical issues in dementia care: systematic qualitative review*	British Journal of Psychiatry (*Psychiatry*)	Clinical ethics: other	Ethical issues	Recommendations for methods
	Thys et al. [52]	BE	*Could minors be living kidney donors? A systematic review of guidelines, position papers and reports*	Transplant International (*Transplantation surgery*)	Clinical Ethics: Transplantation	Ethical recommendations	No
2012	Choo et al. [53]	UK	*Ethical issues and challenges in pressure ulcer research—the research nurses' perspective*	Journal of Tissue Viability (*Dermatology*)	Research Ethics: Nursing	Ethical principles and issues	No
	Dinç et al. [10]	TR	*Trust and trustworthiness in nursing: an argument-based literature review*	Nursing Inquiry (*Nursing*)	Clinical Ethics: Nursing	Ethical arguments	No
	Kanekar and Bitto [54]	US	*Public health ethics related training for public health workforce: an emerging need in the United States*	Iranian Journal fo Public Health (*Public, Environmental and Occupational Health*)	Public Health Ethics	Ethical considerations	Recommendations for training
	Mahieu and Gastmans [55]	BE	*Sexuality in institutionalized elderly persons: A systematic review of argument-based ethics literature*	International Psychogeriatrics (*Psychology, psychiatry, geriatrics*)	Clinical Ethics: Geriatrics	Ethical arguments	No
	Mobasher et al. [56]	IR	*Key ethical issues in pediatric research: islamic perspective, Iranian experience*	Iranian Journal of Pediatrics (*Pedriatrics*)	Research Ethics: Paediatric research	Ethical recommendations and arguments	Recommendations for practice
2011	Droste et al. [57]	DE	*Ethical issues in autologous stem cell transplantation (ASCT) in advanced breast cancer: a systematic literature review*	BMC Medical Ethics (*Medical Ethics*)	Clinical ethics: other	Ethical issues	No
	Dulhunty et al. [58]	AU	*Determining authorship in multicenter trials: a systematic review*	Acta Anaesthesiological Scandinavica (*Anesthesiology*)	Research Ethics: Clinical trial	Ethical considerations in authorship	Recommendations for authorship
	Sofaer and Strech [59]	UK	*Reasons why post-trial access to trial drugs should, or need not be ensured to research participants: a systematic review*	Public Health Ethics (*Public, Environmental and Occupational Health*)	Research Ethics: Clinical trial	Ethical arguments	Recommendations for practice
	Strech and Schildmann [60]	DE	*Quality of ethical guidelines and ethical content in clinical guidelines: the example of end-of-life decision-making*	Journal of Medical Ethics (*Medical Ethics*)	Clinical Ethics: End of Life	Ethical recommendations	Recommendations for practice
	Zwijsen et al. [61]	NL	*Ethics of using assistive technology in the care for community-dwelling elderly people: an overview of the literature*	Aging & Mental Health (*Psychology, psychiatry, geriatrics*)	Clinical Ethics: Geriatrics	Ethical issues	No
2010	Kangasniemi [62]	FN	*Equality as a central concept of nursing ethics: a systematic literature review*	Scandinavian Journal of Caring Sciences (*Nursing*)	Clinical Ethics: Nursing	Ethical concepts	No
	Koelch et al. [63]	DE	*Safeguarding children's rights in psychopharmacological research: ethical and legal issues*	Current Pharmaceutical Design (*Pharmacology and pharmacy*)	Research Ethics: Paediatric research	Ethical issues	No
	Wernow and Gastmans [64]	US	*A Review and Taxonomy of Argument-Based Ethics Literature regarding Conscientious Objections to End-of-Life Procedures*	Christian Bioethics (*Religion, philosophy*)	Clinical Ethics: End of Life	Ethical arguments	Recommendations for practice

JAF (Journal Academic Field), review topics, objects of review and recommendation statuses are the result of our own analysis and subsequent classification. Countries abbreviations: AU: Australia / BE: Belgium / DE: Germany / FI: Finland / IE: Ireland / IR: Iran / MX: Mexico / NL: Netherlands / SP: Spain / TR: Turkey / UK: United Kingdom / US: United States of America

Together with this classification, the *localisation of the citation* in the citing publications using the IMRaD structure was retrieved by two researchers (ZL and HN), partly with the support of an intern. Citations within publications using the IMRaD structure were classified in the following sections: (1) Abstract; (2) Introduction and Background; (3) Methods; (4) Results; (5) Discussion and Conclusions; (6) Limitations or Strengths and Weaknesses; (7) Appendix or Supplements.

Lastly the instances where SREL had been cited in the form of *direct quotations* were documented.

### Synthesis

Once the citations were available and categorised for each SREL, they were introduced in the form of nominal or categorical values in the statistical software *SPSS* (IBM SPSS Statistics for Windows, Version 27). Descriptive statistics (frequencies; range, median, mean; contingency tables) were then applied to the whole dataset. Statistical testing (Pearson’s chi-square) was further applied to investigate the observed different frequencies of methodological citations on the one hand, and thematic citations on the other hand.

## Results

### Systematic reviews of ethical literature

(Supplementary Figures are available in Additional file 1).

#### General characteristics

Table 3 presents an extensive overview of the characteristics of each selected SREL. Selected SREL were published between 2010 and 2015, with a peak in 2014 (*n* = 10). SREL first authors came from Europe (*n* = 19), the USA (*n* = 5), Australia (*n* = 3), Iran (*n* = 2), Turkey (*n* = 1), and Mexico (*n* = 1).

#### Review topics

As shown in Table 3, our sample of SREL shows a relative balance between reviews dealing with research ethics (*n* = 14) and clinical ethics (*n* = 15). One SREL dealt with both clinical and research ethics and another one with Public Health Ethics. Among the reviews within research ethics, clinical trials (*n* = 5) and pediatric research (*n* = 3) are the most common topics. Among the reviews addressing issues belonging to clinical ethics, geriatrics (*n* = 4), nursing (*n* = 3), and end of life (*n* = 3) are the most frequent subjects.

#### Review objects

As displayed in Table 3, most selected SREL (*n* = 14) review ethical issues, topics, or dilemmas specific to a given situation, e.g., ethical issues in dementia care or in the use of assistive technologies for elderly patients. A third of the SREL (*n* = 10) review literature providing arguments for deciding on an ethically sensitive topic, e.g., disclosure of incidental findings arising in genetic studies. Ethical guidelines or recommendations are the objects of review of another third of the selected SREL (*n* = 10), e.g., living organ donation among minors. Some SREL (*n* = 5) focus on literature discussing ethical principles, values or norms, e.g. concept of equality or moral distress in nursing. A few SREL review other objects that include: existing ethics support mechanisms; ethical tools such as institutional bodies, frameworks, educational programs, policies; ethics-related instruction in schools and programs of public health. Since SREL could have more than one object of review, the total number of review objects (*n* = 42) does not correspond to the total number of SREL (*n* = 31).

#### Journal academic fields (JAF)

Table 3 also reveals that most selected SREL were published in journals belonging to the fields of *Medical Ethics* (*n* = 5) and *Nursing* (*n* = 5). 20 SREL were published in different journals across various medical fields (e.g., Public Health, Psychology). One SREL was published in a journal belonging to the philosophical/religious field.

#### Recommendation status

Thirteen of the 31 selected SREL issued ethical recommendations: 7 recommended changes in practice and 6 recommended other changes, for instance changes in methodology or in research focus (see Table 3).

### Citations

#### General characteristics

**Fig. 1:**
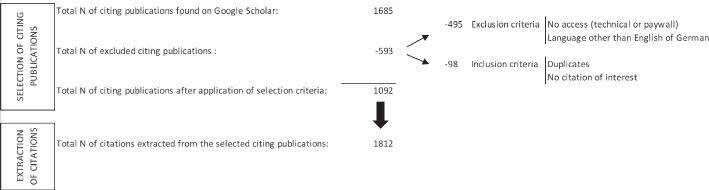
Selection process of the citing publications and citation extraction

As represented in Fig. 1, the number of *citing publications* retrieved on Google Scholar in the given timeframe was 1685 (range from 3 to 224; mean: 52,6; median: 40.5). At the end of our two-step selection process, a total of 593 documents were excluded, leaving 1092 citing publications ready for analysis. Since some publications cite more than once the SREL under consideration, we eventually reached a total of 1812 *citations* to analyse (range from 1 to 303 citations per SREL; mean: 58,4; median: 27). Only 3% of all citations (*n* = 62) are direct quotations from the original SREL. Ninety percent of all citations are of *thematic* nature (*n* = 1623), the remaining 10% are *methodological* (*n* = 189).

**Fig. 2:**
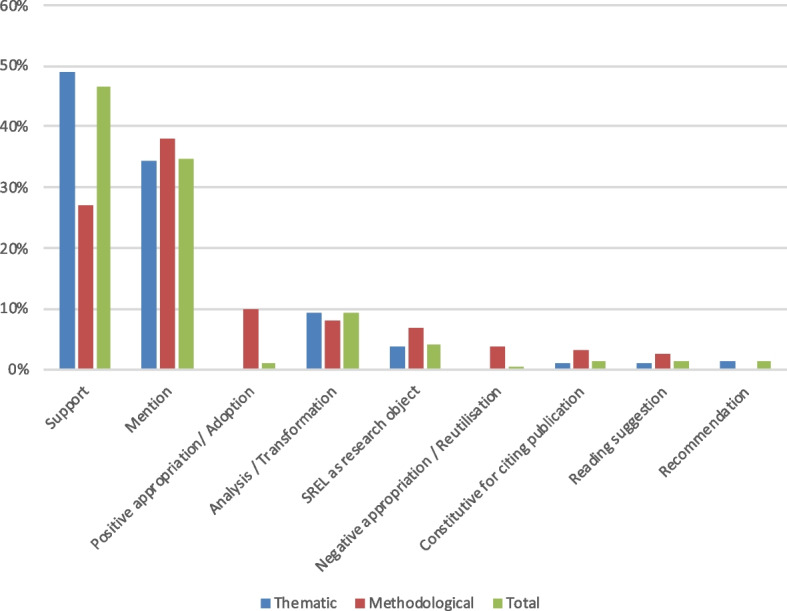
Relative percentages of citations according to the citation type. Legend: Total citations *n* = 1812; thematic citations *n* = 1623; methodological citations *n* = 189

As shown in Fig. 2, citations taken as a whole sample are mostly used to *support* a particular statement, without further discussion or transformation (“support”, 47%, *n* = 846). The second most common function of citations, indistinctive of their nature, is the *mention* of the SREL without further specification (“mention”, 35%, *n* = 628). Within thematic citations, nearly 50% (*n* = 795) are supporting a statement while 25% of methodological citations (*n* = 51) are used for the same purpose. Proportionally methodological citations are more often used as mere mentions (38%, *n* = 72) and sometimes indicate a positive (10%, *n* = 19) or negative appropriation of methods (4%, *n* = 7).

**Fig. 3:**
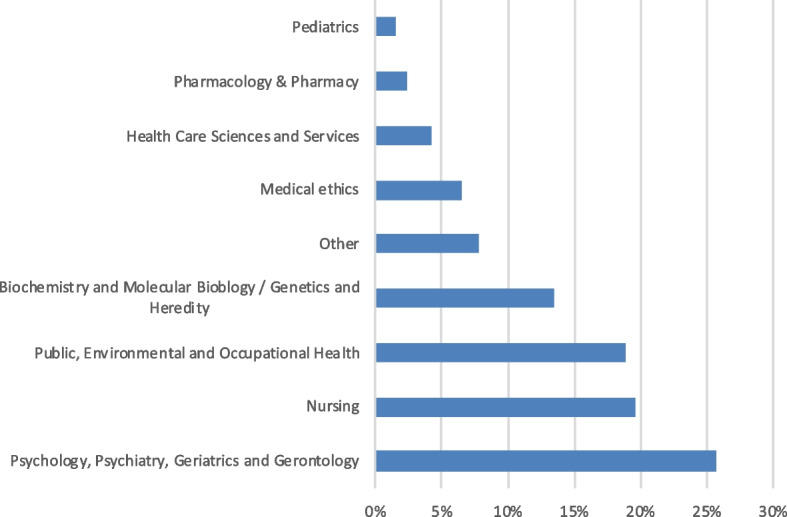
Proportions of citations according to the journal academic fields of the 31 selected SREL. Legend: Total citations *n* = 1812

As shown in Fig. 3, most citations are retrieved from the selected SREL that were published in journals belonging to the academic fields of *Psychology*, *Psychiatry*, *Geriatrics*, and *Gerontology* as well as *Nursing*.

#### Citations according to the nature of the citing publications

Nearly all retrieved citations (96%, *n* = 1753) are located in scientific publications: empirical investigations (39%, *n* = 704), theoretical articles (25%, *n* = 461), systematic reviews (14%, *n* = 252), reviews (e.g., narrative or scoping) (9%, *n* = 161), methodological investigations (7%, *n* = 124), letters (1%, *n* = 23), editorials (1%, *n* = 17), and scientific reports (1%, *n* = 11). The remaining citations (4%, *n* = 59) are found in conference proceedings (1.6%, *n* = 30), newspaper articles (< 1%, *n* = 11), patient information (< 1%, *n* = 6), guidelines or recommendations (< 1%, *n* = 3), teaching material (< 1%, *n* = 3), blogs (< 1%, *n* = 2), and others (< 1%, *n* = 4).

**Fig. 4:**
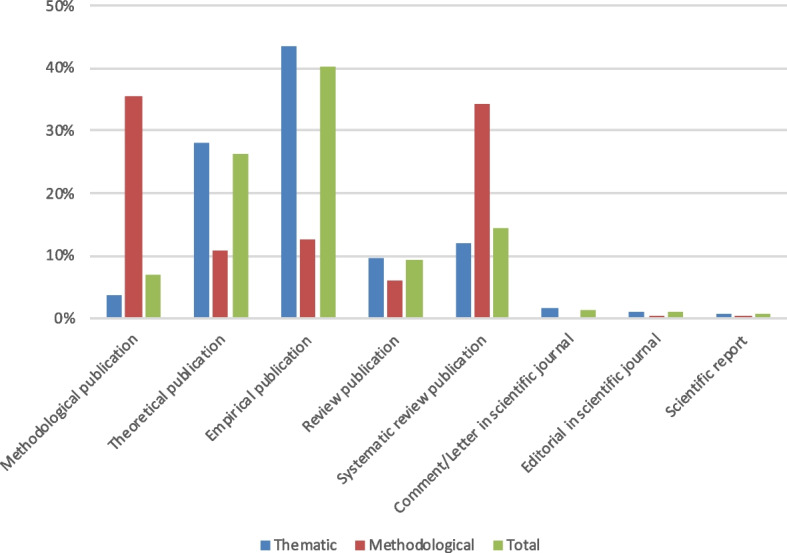
Relative percentages of citations according to the nature of the citing publications. Legend: Total citations in scientific publications only (*n* = 1753): thematic citations *n* = 1569; methodological citations *n* = 184

As shown in Fig. 4, within scientific publications, most thematic citations are found in empirical (43%, *n* = 680) and theoretical publications (28%, *n* = 441). Most methodological citations are found in methodological publications (35%, *n* = 65) and in systematic reviews (34%, *n* = 63).

In our sample, thematic citations are mostly used to support statements across the different publication types (22%, *n* = 346 in empirical publications; 13%, *n* = 213 in theoretical publications). Thematic citations are also often used as mere mentions (16%, *n* = 262 in empirical publications; 8%, *n* = 131 in theoretical publications). Thematic citations indicating some form of analysis or transformation of content are predominantly found in theoretical (4%, *n* = 66) and empirical scientific publications (3.5%, *n* = 55). For more details on thematic citations, see Figure S1 in Additional file 1.

Methodological citations often appear to be mentions across the different publication types (e.g., 16%, *n* = 30 in methodological publications; 12,5%, *n* = 23 in systematic reviews). They are also used as support (e.g., 8.6%, *n* = 16 in methodological publications; 8.1%, *n* = 15 in systematic reviews). In systematic reviews, and to a minor extent in reviews, methodological citations also indicate an appropriation or an adaptation of the method (7.6%, *n* = 14) as well as the use of the SREL as a research object (2.7%, *n* = 5 in systematic reviews; 2.1%, *n* = 4 in methodological publications). For more details on methodological citations, see Figure S2 in Additional file 1.

The observed high proportions of methodological citations in methodological and systematic reviews publications as well as the high proportions of thematic citations indicating an analysis or transformation of content in conceptual publications were confirmed as statistically significant in this sample through Pearson’s chi-square tests (for thematic citations: *χ*^2^ = 601.8; df = 112; Asymp. Sig. = 0,000; for methodological citations: *χ*^2^ = 507.3; df = 126; Asymp. Sig. = 0.000; α = 0.05).

#### Citations according to their localisation within the IMRaD structure

**Fig. 5:**
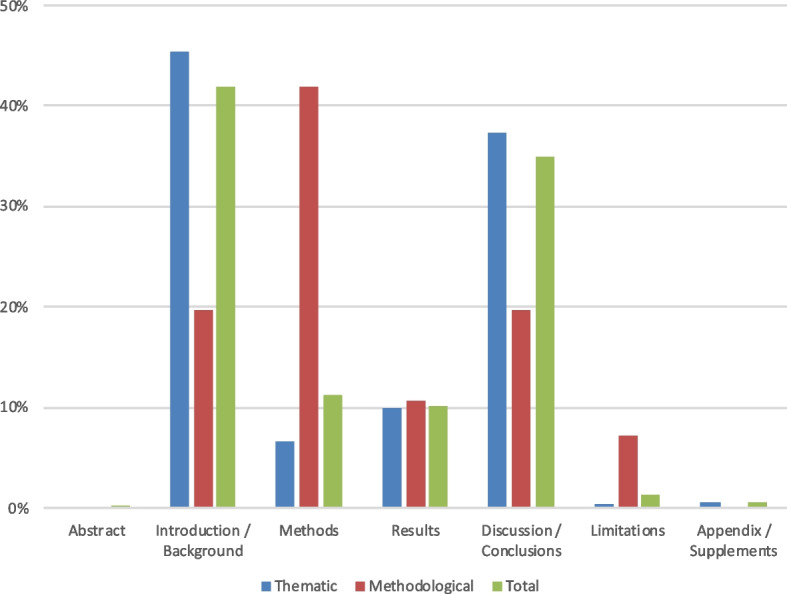
Relative percentages of thematic and methodological citations according to their localisation (IMRaD structure). Legend: Publications using the IMRaD structure only (*n* = 833); thematic citations *n* = 721; methodological citations *n* = 112

About half of the identified citations (*n* = 833) were retrieved in publications using the IMRaD structure. As illustrated in Fig. 5, most of these citations were found in the *Introduction* / *Background* (42%) and the *Discussion/Conclusion* (35%) of these publications. Accordingly, 45% of all thematic citations are found in the *Introduction* and 37% in the *Discussion*. Methodological citations are mostly present in the *Methods* Sect. (42%).

When looking at the specific nature of the citations, we see that, across the different IMRaD sections, most thematic and methodological citations are used to support a statement, indicating that their content is not discussed or further analysed. Furthermore, some methodological citations found in the methods section (*n* = 11, 10%) indicate either an appropriation or an adaptation of the method described in the SREL.

Among thematic citations, mentions (*n* = 144, 20%) or support (*n* = 156, 22%) were mainly found in the *Introduction/Background* section, followed by the *Discussion/Conclusions* section (mentions: *n* = 84, 11%; support: *n* = 135, 19%); considerably fewer occurred in the *Methods* (mention: *n* = 11, 1.5%; support: *n* = 23, 3%) and *Results* (mention: *n* = 14, 2%; support: *n* = 33, 4.5%) sections. Thematic citations indicating analysis or transformation were mainly found in the *Discussion/Conclusion* sections (*n* = 34, 5%). For more details on thematic citations according to publication section, see Figure S3 in Additional file 1.

Among methodological citations, mentions (*n* = 14, 12,5%), support (*n* = 13, 12%), and citations indicating a positive appropriation (*n* = 11, 10%) were mainly found in the *Methods* section. The latter were also found, to some extent, in the discussion section (*n* = 5, 4,5%). An overview of these findings can be found in Figure S4 in the Additional file 1.

Most of these observations are statistically significant following Pearson’s chi-square tests, i.e., methodological citations indicating mention, support and object of research are more present in *Methods*; thematic citations indicating that SREL were taken as objects of research are found in *Methods*; and thematic citations indicating analysis/transformation are dominant in *Discussion / Conclusion* (for thematic citations: *χ*^2^ = 431.5; df = 56; Asymp. Sig. = 0.000; for methodological citations: *χ*^2^ = 408.6; df = 63; Asymp. Sig. = 0.000; α = 0.05).

#### Citations according to the review object of the systematic review of ethical literature

**Fig. 6:**
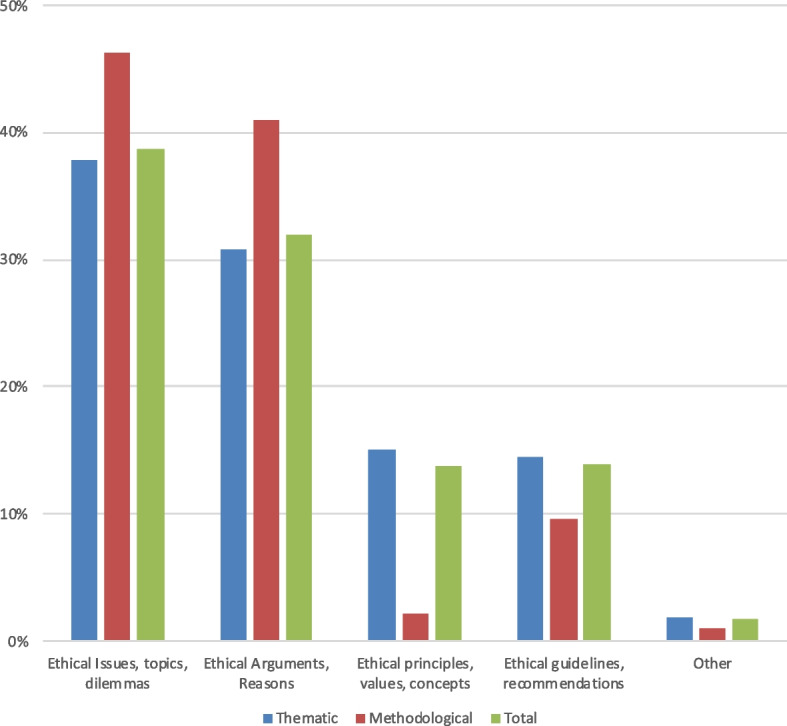
Relative proportions of the citations according to the object of review of the SREL. Legend: Total citations *n* = 1812; thematic citations *n* = 1623; methodological citations *n* = 189

As illustrated in Fig. 6, most citations come from SREL that had reviewed ethical issues, topics or dilemmas (39%, *n* = 702) followed by SREL on ethical arguments or reasons (32%, 578). The remaining citations are evenly distributed between SREL that reviewed ethical principles, values or concepts (14%, *n* = 249) and ethical guidelines and recommendations (14%, *n* = 252). Thematic citations follow this general distribution while methodological citations frequently come from SREL that reviewed ethical issues, topics and dilemmas (46%, *n* = 87) as well as ethical arguments or reasons (41%, *n* = 77).

**Fig. 7:**
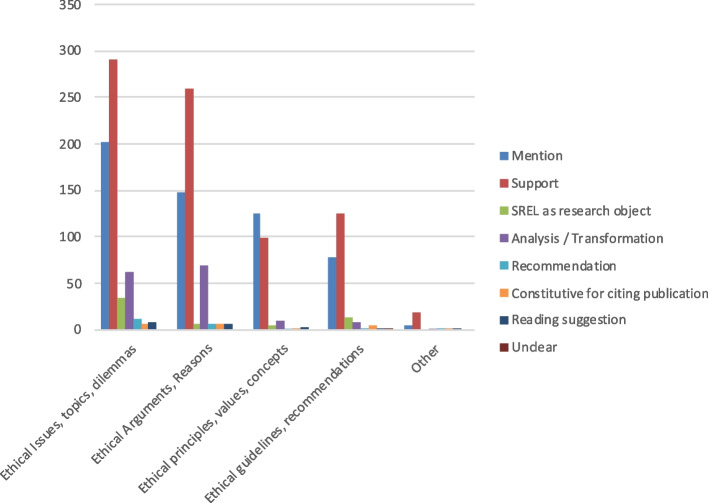
Number and nature of thematic citations according to the objects of review of the SREL. Legend: Total thematic citations *n* = 1623

As shown in Fig. 7, thematic citations indicating analysis or transformation mostly come from SREL reviewing ethical arguments (*n* = 69, 4%) and ethical issues, topics, and dilemmas (*n* = 63, 3.8%). Otherwise, support citations dominate among all types of SREL, but also mention citations, while the remaining citation types do not differ significantly.

**Fig. 8:**
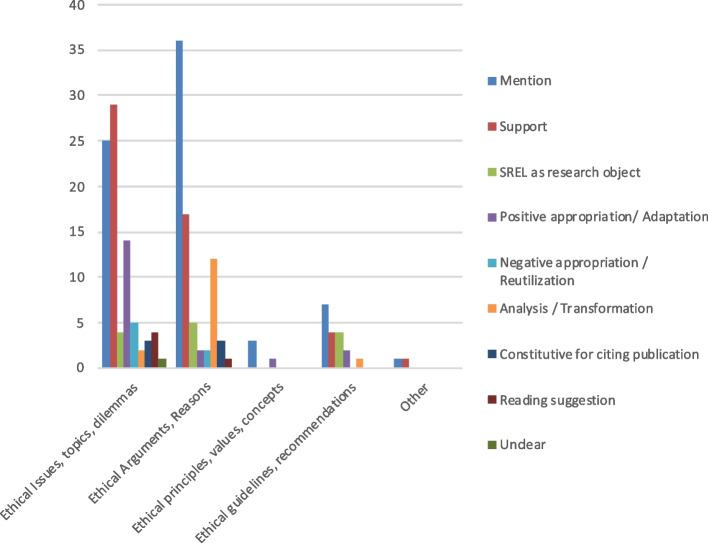
Number and nature of methodological citations according to the objects of the SREL. Legend: Total methodological citations *n* = 189

As shown in Fig. 8, methodological citations tend to be mere mentions or to be used as support, especially when they are citations from SREL on ethical issues and ethical arguments (*n* = 28, 15%). Methodological citations indicating a form of appropriation are more common when they cite SREL that focused on ethical issues, topics and dilemmas (*n* = 13, 7%). Methodological citations indicating an analysis or a transformation were citing SREL that reviewed ethical arguments or reasons (*n* = 12, 6%).

#### Citations according to the recommendation status of the systematic review of the ethical literature

Most citations (64%, *n* = 1164) were retrieved from SREL that did not issue recommendations. Among the citations of SREL that issued recommendations (36%, *n* = 648), the majority came from reviews that issued *ethical* recommendations (24%, *n* = 434) and the rest from reviews that issued recommendations of another nature (12%, *n* = 214). As methodological citations are evenly distributed across the different categories, they are also proportionally more commonly made from SREL that issued ethical recommendations (34%, *n* = 65) than thematic citations (23%, *n* = 369). An overview of these findings can be found in the Figure S5 in Additional file 1.

Among the citations of those SREL that issued recommendations (36%, *n* = 648), 6 citing publications issued themselves some recommendations. A limited qualitative analysis revealed that, in most cases (*n* = 4), the recommendations entailed in the citation were covered by the SREL referenced in the text. In two occurrences, we could see that the recommendation entailed in the citation was only partially covered by the SREL.

## Discussion

In order to understand the actual impact of systematic reviews of ethical literature, we decided to investigate the way SREL are used and referenced in the scientific literature. To this end, we identified the citations of a sample of 31 SREL, reaching a total sample of 1812 citations (ranging from 1 to 303 citations per SREL). We then proceeded to a two-fold analysis, qualitative and quantitative, of the SREL citations, examining the specific context in which each citation occurs. Our systematic analyses of SREL citations provide an unprecedented preliminary insight into the functions of SREL citations that will hereafter be discussed in more detail.

Taking the sample as a whole, it appears that the selected SREL were primarily cited to *support* specific statements on, for instance, ethical arguments, issues or principles (*n* = 846, 47% of all citations; *n* = 795 thematic citations, *n* = 51 methodological citations). When citations indicate such a function of *support*, it means that SREL are referenced to establish the value or the credibility of a specific statement. This can, to some extent, be related to the fact that, in our sample, the most cited SREL are the ones dealing with ethical issues, topics or dilemmas (39% of all citations) and ethical arguments or reasons (32% of all citations). Moreover, our analysis of the citations according to their localisation in the IMRaD structure showed that these *support* citations occurred mainly in the introduction and in the discussion, as one would expect. This way, the evidence provided in SREL is used in order to ground the existence, the relevance or even the reliability of a statement. Such use of the data generated through SREL de facto fulfills one of the major purposes of systematic reviews.

In our study, the second most common use of citations from SREL indicate *mentions* (*n* = 628, 35% of all citations; *n* = 556 thematic citations, *n* = 72 methodological citations; see Fig. 2). The main goal of such *mentions* is merely to indicate the existence of a SREL, possibly orienting the readers. Our qualitative content analysis of the citations unveils the sometimes very unspecific character of such mentions. We indeed noticed that several citations could have interchangeably mentioned any other publication on the given topic as the specificities of the SREL actually cited were not relevant to the text. For instance, we sometimes observed the reference to ethical principles well known in the field but that are not the direct result of the SREL cited. It appears that superficial citing is by no means exclusive to SREL. Indeed what we designated as mere “mentions” in this study seems to correspond to what is referred to under the categories of “perfunctory” or “superficial” in citationology and that is commonly observed in many scientific fields [20]. Although the number and the nature of citations alone cannot and should not be taken as sole indicator of a meaningful use of SREL, we were able to see that mentions as common citation practice depart to a great extent from the expected impact of SREL, rather characterized by thematic specificity and methodological rigor.

Citations indicating *Analysis/Transformation* were quite rare in our sample (*n* = 168, 9% of all citations; *n* = 153 thematic; *n* = 15 methodological—see Fig. 2), and were, logically, mainly encountered in the discussion section. This data indicates that, in our sample, SREL were only occasionally the starting point of an ethical discussion, as it is arguably more common in theoretical papers, especially philosophical-ethical papers where concepts are discussed in more depth. This observation can possibly be explained by a still relatively low awareness about SREL and their unique value as systematic overview on arguments, issues or concepts on a topic. Another explanation could be the opinion according to which syntheses produced by SREL are too general or superficial to become the subject of an in-depth debate. It could be that researchers sharing this view rather use SREL as a way to identify single publications to then engage in-depth with these, eventually citing these specific publications rather than SREL in their own work.

Although our sample is dominated by thematic citations, methodological citations still constitute 10% of all citations (*n* = 189). Given the intended purpose of a SREL to inform primarily on ethical issues, the fact that a tenth of the selected citations have a methodological nature is noteworthy and seems to indicate that SREL are substantially cited for methodological purposes. Acknowledging the possible contingency linked to our sample, we can still try to explain this phenomenon. Indeed, in a context where SR method is rather new in bioethics and where formulated method papers or manuals are still rare, it is quite understandable that a SREL aiming to be published will refer to an already successfully published SREL–or reviews in ethics in general. In this sense, SREL can be cited either to justify (*support*, *n* = 16 methodological citations) or modify one’s own methodological approach (*positive or negative appropriation*, *n* = 26 methodological citations). Similarly, methodological papers that deal precisely with the SREL method will naturally cite specific SREL as examples (*n* = 65 methodological citations, all uses). These hypotheses can further be supported by two of our study findings: (1) methodological citations were more common within the methods section; (2) methodological citations were sometimes indicating an *object of research* (*n* = 13, 7%) in which SREL could have been part of a methodologically oriented meta-review.

Our analysis of the nature of the citing publications from which the selected citations originate show that most citations were found in *empirical publications* (43% of all citations). A smaller share of citations was found in *theoretical publications*, including conceptual papers (28% of all citations). A possible explanation for this observation could be that our sample included SREL that appear to have specific thematic relevance for empirical contributions, e.g., the SREL from Mikesell et al. [30] was referenced by 150 publications presenting empirical community-engaged research. Although we acknowledge that this could be different in other samples, it is possible that, perhaps contrary to a first intuition, SREL also constitute an ethical input that can have implications specifically for empirical research. In the same line, we can observe that, although *Medical Ethics* is the predominant journal academic field among our 31 selected SREL, there is comparatively only a small proportion of citations of these articles. While this could be a contingent piece of data, it could also reflect the fact that ethics is not restricted to a single academic field but tend to gain meaning in many different fields, including empirically based research.

Lastly, our results seem to challenge an initial assumption according to which SREL are expected to serve as evidence for guidelines and similar recommendations, see for instance [19]. The use of SREL as input for guidelines indeed appears to be only marginally represented in our sample. First, only 36% of the 31 selected SREL include some form of recommendation. Second, just 3 out of the 1092 included citing publications were identified as guidelines or recommendations. Third, in our qualitative analysis, only 22 thematic citations were categorised as “recommendations”. Our data furthermore challenge the hypothetical concern linked to using SREL to justify ethical recommendations with practical implications [5]. On the basis of our findings, it seems instead appropriate to consider the function of *supporting statements* by SREL as the most relevant, the majority of which do not—and need not—relate to guidelines or the justification for recommendations. If the “evidence function” of SREL for guidelines and HTA reports were to be set as an essential purpose of SREL, adjustments would be necessary. These could, for instance, include specific education on this aspect for ethics researchers or active involvement in the development of (ethical) guidelines, related methods and processes, see for example [15].

### Limitations

To our knowledge, our study is the first to systematically investigate the actual uses of SREL through their citations in published documents. While we actively sought to avoid preventable biases, we do acknowledge the possibility of limitations and detail them hereafter.

In our study design, the decision to analyse SREL uses through their citations inevitably led to the exclusion of all instances in which SREL are used for purposes different than publications, e.g., input for clinical practice or for teaching purposes. However, citations are an essential feature of publication uses, especially in the academic field. Furthermore, identifying potential users of SREL could turn out to be an uncertain endeavour. Therefore, and despite the limitations it entails, we consider that the adopted approach was a first realistic step towards gaining insights into SREL uses.

The 31 SREL from which we searched and analysed the citations is a sample drawn from a selection we previously established and published in a comprehensive meta-review. Within this initial list, we decided to focus on reviews of normative nature that were published between 2010 and 2015. We decided to use this sample because it appeared to us that SREL published in this timeframe were the most suited for this analysis. On the one hand, at that time, SREL already had a relatively sound methodological basis. On the other hand, and since our citations search happened in 2020, it allowed a reasonable period of 6 to 10 years for the SREL to enter the citation cycle. However, characteristics such as publishing journals or journal academic fields of these SREL are inevitably contingent and the fact that we restricted our analysis to SREL published between 2010 and 2015 could have led to some form of biases on this regard. Such characteristics are likely to depend significantly on factors that could not be examined in detail in the context of this study, e.g., relevance of SREL topics, SREL legacy in the ethical debate, possible “citation cartels”.

For the citation search, we decided to use Google Scholar as it appeared to be the best suited search engine to retrieve a variety of publication types besides scientific ones. Using Google Scholar still carries some limitations due to technical issues (e.g., inactive links to citing publications) and imprecisions (e.g., absence of reference to the SREL of interest in the publication). We had to cope with these limitations during the selection phase and this could have led to some form of bias.

In the process of citation selection, we decided to focus on citing publications that were published in the languages fluently read by all researchers involved in the study, i.e., English and German. As a result, 126 citing publications were excluded on this basis. This could constitute a selection bias due to the exclusion of some marginally represented geographical areas. In the same selection step, we excluded all documents that were not openly accessible or not accessible through our institutional credentials. The rationale for this decision was mainly linked to the resources available for this project. Although we kept a systematic track of the exclusions linked to these selections, we cannot completely rule out the possibility that the exclusion of about a quarter of the initial hits (citing publications) could have led to some biases in our sample, data selection and analysis. However, and in view of the clear trends that crystallized in our sample (e.g., on *mentions* and *support* functions), it can be considered unlikely that the analysis of these excluded citing publications would have led to radically different trends. Still, in case the functions and citing publication types that rarely occurred in our sample were coincidentally more common among the excluded citing publications, we cannot completely rule out a residual risk of bias linked to these exclusions.

We performed data search, selection, analysis, and synthesis in a systematic way and we consistently applied the four-eyes principle, i.e., for each step of the analysis, at least two researchers independently processed the data, compared their results and, if necessary, discussed them to reach consensus. Still, the authors’ backgrounds and familiarity with a given topic may be relevant to understand the specific perspective adopted during qualitative analyses, including its potential for biases. In our study, the researchers’ backgrounds appear to have ensured the appropriateness of the categorisation of the citations during the first qualitative step of our research. Indeed, the philosophical and bioethical backgrounds of most authors (HN, ZL, AS, and MM) helped the contextualization of the citations and the development of adequate categories. The extensive knowledge on systematic reviews methods in ethics and related meta-research of HN and MM enabled to frame the results and to determine their relevance. While working under the close supervision of HN and MM, the two researchers who performed most of the qualitative analyses (NR and ZL) did not have previous experience with SREL. This allowed for the qualitative investigation to be completed with a low theoretical preconception which may reduce the possibility of bias. Nevertheless, despite the care we put in data gathering and processing, we cannot completely exclude the possibility of some residual subjective bias during data analysis and synthesis.

## Conclusions

Our investigation provides evidence about the role of SREL in scientific literature. On the one hand, our analysis showed that SREL are mostly used as references to provide support or credibility to specific statements about ethical issues. Although these references sometimes appear to be imprecise, SREL seem to play an important role in providing state-of-the-art summaries of available reflections to pursue the scientific discussion on a given topic. On the other hand, SREL appear to be used as methodological orientation, e.g., for the design of a SR or for the implementation of ethical empirical research. In the first case, SREL seem to fill a persisting gap caused by the (perceived?) lack of methodological guidance to perform SREL.

Besides the provision of insights into SREL uses, the present study also provides new methodological tools and categorisations that could be useful for future research. The developed study design could, for instance, be used as a basis for a future similar study with a purposive sample or in different scientific disciplines. Such future research could further contribute to the testing and improvement of the reliability of the categories. It would also allow to determine whether our results indicate features specific to systematic reviews of ethical literature or features common in systematic reviews conducted in other disciplines, e.g., medicine.

From a practical standpoint, examining further SREL uses could moreover be helpful in order to improve the processing of SREL. A future valuable analysis could consist in checking more closely whether SREL citations are meaningful, also in terms of specific content of each citation with regards to the cited text. This could mean, for instance, to examine thoroughly whether citations are actually covered by the statements entailed in the SREL. Our original citation analysis may well provide a basis for this, since each of the citations we have identified could be subjected to closer scrutiny in a secondary data analysis.

Ultimately our study may also constitute a valuable first input towards the investigation of the contrast between theoretical debates about SREL purposes and SREL actual uses. Taking stock of the discrepancies between both areas could further encourage to search ways to align both debates in a constructive way. This could go in two different directions: either towards an adjustement of the expected value from a theoretical point of view, or towards practical changes in the conduct and especially in the presentation of SREL results, in order to improve its output for potential users.

### Supplementary Information

**Additional file 1: **Supplementary figures which supplement the figures in the manuscript.

## Data Availability

The SPSS file containing the analysed data is available from the corresponding author upon reasonable request.

## Abbreviations

HTA: Health Technology Assessment
PRISMA: Preferred Reporting Items for Systematic Reviews and Meta-Analyses
SR: Systematic review
SREL: Systematic review of ethical literature
