# Correction: What can the citations of systematic reviews of ethical literature tell us about their use?—an explorative empirical analysis of 31 reviews

**DOI:** 10.1186/s13643-023-02368-1

**Published:** 2023-11-08

**Authors:** Hélène Nobile, Natali Lilie Randjbar Moshtaghin, Zoë Lüddecke, Antje Schnarr, Marcel Mertz

**Affiliations:** 1 Institute for Ethics, History and Philosophy of Medicine, Hannover Medical School, Carl-Neuberg-Str. 1, 30625 Hannover, Germany; 2 Institute for the History of Medicine and Medical Ethics, Faculty of Medicine and University Hospital, University of Cologne, Joseph-Stelzmann-Str. 20, Geb. 42, 50931 Cologne, Germany

**Correction: Syst Rev 12, 173 (2023)**

10.1186/s13643-023-02341-y

Following publication of the original article [1], the author reported that the abstract and keywords were missing.

**Abstract**

**Background** Systematic reviews of ethical literature (SREL) aim at providing an overview of ethical issues, arguments, or concepts on a specific ethical topic. As SREL are becoming more common, their methodology and possible impact are increasingly subjected to critical considerations. Because they analyse and synthetise normative literature, SREL are likely to be used differently than typical systematic reviews. Still, the uses and the expected purposes of SREL were, to date, mainly theoretically discussed. Our explorative study aimed at gaining preliminary empirical insights into the actual uses of SREL.

**Methods** Citations of SREL in publications, both scientific and non-scientific, were taken as proxy for SREL uses. The citations of 31 published SREL were systematically searched on Google Scholar. Each citation was qualitatively analysed to determine its function. The resulting categorisation of SREL citations was further quantitatively investigated to unveil possible trends.

**Results** The analysis of the resulting sample of SREL citations (*n* = 1812) showed that the selected SREL were mostly cited to support claims about ethical issues, arguments, or concepts, but also to merely mention the existence of literature on a given topic. In this sample, SREL were cited predominantly within empirical publications in journals from various academic fields, indicating a broad, field-independent use of such systematic reviews. The selected SREL were also used as methodological orientations either for the conduct of SREL or for the practical and ethically sensitive conduct of empirical studies.

**Conclusions** In our sample, SREL were rarely used to develop guidelines or to derive ethical recommendations, as it is often postulated in the theoretical literature. The findings of this study constitute a valuable preliminary empirical input in the current methodological debate on SREL and could contribute to developing strategies to align expected purposes with actual uses of SREL.

**Keywords**

Systematic review, literature review, meta-research, ethics, citation practice

The original article [1] has been corrected.
