# Supplementary material for: What can the citations of systematic reviews of ethical literature tell us about their use?—an explorative empirical analysis of 31 reviews
Source: Syst Rev. 2023 Sep 23;12:173. doi: 10.1186/s13643-023-02341-y (PMC10517474; doi:10.1186/s13643-023-02341-y)
Supplement: Supplementary file 1 — Additional file 1: Supplementary figures which supplement the figures in the manuscript. [file 13643_2023_2341_MOESM1_ESM.docx]

# SUPPLEMENTARY FIGURES

Figure S1: Nature of thematic citations according to the type of citing publications, scientific publications only, 1 unclear citation excluded (n=1569)

Figure S2: Nature of methodological citations according to the type of citing publications, scientific publications only, 1 unclear citation excluded (n=184)

Figure S3: Nature of thematic citations according to their localisation in the publications using IMRaD only (n=721)

Figure S4: Nature of methodological citations according to their localisation in the publications using IMRaD only (n=112)

Figure S5: Proportions of methodological and thematic citations according to the recommendation status of the SREL they cite (Total citations n=1812: Thematic citations n=1623; Methodological citations n= 189).
